# Endometrial estrogen and progesterone receptor activity in unusual chronology of pubertal development: a case report

**DOI:** 10.61622/rbgo/2025rbgo63

**Published:** 2025-10-21

**Authors:** Michelly Siqueira-Souza, Carolina Chicharo Vivas, Jéssica Couto dos Anjos, Gabrielle Barbosa Anelli Morau, Júlia Kefalás Troncon, Lucianna Fonseca Barreto, Carolina Gennari Verruma, Rosana Maria dos Reis

**Affiliations:** 1 Universidade de São Paulo Faculdade de Medicina de Ribeirão Preto Departamento de Ginecologia e Obstetrícia Ribeirão Preto Brazil Departamento de Ginecologia e Obstetrícia, Faculdade de Medicina de Ribeirão Preto, Universidade de São Paulo, Ribeirão Preto, Brazil.; 2 Universidade de São Paulo Faculdade de Medicina de Ribeirão Preto Departamento de Patologia Ribeirão Preto Brazil Departamento de Patologia, Faculdade de Medicina de Ribeirão Preto, Universidade de São Paulo, Ribeirão Preto, Brazil.

**Keywords:** Menarche, Uterine hemorrhage, Hysteroscopy, Endometrium, Biopsy, Puberty

## Abstract

An 8-year-old girl presented with vaginal bleeding without puberty signs. During investigation at 10 years old, the patient presented with advanced bone age, prepubertal hormone levels and internal genitalia. Hysteroscopy and vaginoscopy at 10 years and 10 months of age revealed endometrial proliferation despite infantile genitalia. Histopathology showed positive receptors for estrogen and progesterone. This is the first report of endometrial estrogen and progesterone receptor activity in this context. Despite normal serum estradiol, the findings suggest a local action of estrogen. Follow-up until 11 years of age showed progressive development of secondary sexual characteristics. This case report emphasizes the need to consider isolated vaginal bleeding, without the development of secondary sexual characteristics, as a result of endometrial hypersensitivity to low estrogen levels during pubertal development, which may alter the chronology of pubertal development in girls.

## Introduction

Puberty is a critical period of development characterized by the acquisition of sexual maturity, regulated by the activation of the hypothalamic-pituitary-gonadal (HPG) axis.^(1)^ In humans, the age of puberty onset varies according to multiple factors, such as genetic background, ethnicity, and gender.^(1,2)^ The first pubertal signs are generally observed between the ages of 8 and 13 years in girls,^(2,3)^ with the appearance of mammary buds (thelarche), which corresponds to Tanner stage 2 of breast development.^(4,5)^

Approximately six months after thelarche begins, the growth of pubic hair, named pubarche, will typically occur.^(6)^ Finally, menarche occurs, marking the girls first menstrual period. It typically happens approximately six months after the peak height velocity has been reached and is triggered by increased levels of follicle-stimulating hormone (FSH) and luteinizing hormone (LH).^(7)^ During puberty, the uterine endometrium undergoes cycles of proliferation and regression due to fluctuating plasma estradiol levels. Once sufficient endometrial growth has been achieved, the subsequent withdrawal of estrogen triggers the first menstrual bleeding.^(6)^

The occurrence of bleeding before thelarche is very rare.^(8,9)^ When it occurs, either as an isolated or recurrent event in prepubertal girls without secondary sexual characteristics, it is defined as prepubertal vaginal bleeding or benign prepubertal vaginal bleeding.^(8,10)^ These conditions are considered as an incomplete form of puberty since no other signs of puberty are present.^(8)^

Incomplete puberty, such as premature pubarche or thelarche, is usually considered a variant of normal puberty, but it may be caused by an underlying pathology that should be sought, or a sign of a central precocious puberty that will develop in the future.^(11)^ However, there is no consensus that isolated menarche represents a disorder of pubertal development, as menarche is typically considered a late pubertal event that occurs only after full activation of the HPG axis.^(12)^ Nonetheless, it has been suggested that isolated menarche may result from a partial and transient activation of this axis, sufficient to stimulate endometrial proliferation and withdrawal bleeding, without triggering other secondary sexual characteristics.^(13)^

Given the ongoing debate regarding the classification of isolated menarche and its clinical implications, we present a rare case of a young girl who presented with cyclical vaginal bleeding mimicking menstruation, occurring before thelarche and pubarche, and in the absence of other signs of puberty. This unusual presentation poses a diagnostic challenge and highlights the importance of careful evaluation of prepubertal bleeding in the absence of other secondary sexual characteristics. This case report was approved by the Research Ethics Committee of the Hospital das Clínicas de Ribeirão Preto – Universidade de São Paulo (CAAE: 79258424.4.0000.5440).

## Case description

A Caucasian girl presented with irregular vaginal bleeding at the age of 8 years and 7 months. The bleeding episodes lasted approximately two days, with small to moderate volume, associated with mild dysmenorrhea and headache. However, she had no initial breast or pubic hair development, and secondary sexual characteristics were classified as Tanner M1P1, with prepubertal external genitalia. As a personal history, she had dysautonomic syndrome and was under treatment with coenzyme Q10 (CoQ10) (400 mg/day) about two years before the first bleeding. Physical examination revealed that her growth curve was at the 75th percentile, which is consistent with the familial canal. Table 1 illustrates the evolution of pubertal development of the patient, during 2 years of follow-up.

**Table 1:** Evolution of pubertal development of the patient, during 2 years of follow-up

Age (Years/Months)	10y2m	10y4m	10y8m	10y10m	10y11m	11y2m	11y9m
Vaginal Bleeding	Yes	Yes	Yes	Yes	Yes	Yes	Yes
Bleeding Regularity	Irregular since 8y7m	Irregular	Irregular	Irregular	Irregular	Irregular	Irregular
Bleeding volume	Light to moderate	Light to moderate	Light to moderate	Light to moderate	Light to moderate	Light to moderate	Light to moderate
Pubertal Development	M1P1	M2P2	M2P2	M2P2	M2P2	M3P3	M3P3
External Genitalia	Prepubertal	Prepubertal	Prepubertal	Prepubertal	Prepubertal	Prepubertal	Prepubertal
Weight (Kg)	33.60	33.70	34.70	-	37.40	-	43.20
Height (m)	142.50	145.00	145.00	-	148.00	-	153.00
IMC (Kg/m^2^)	16.50	16.00	16.50	-	17.10	-	18.50
Axillary Odor	-	-	Yes	-	-	-	-
Bone Age (Years)	12	-	-	-	-	12	-
Exams	Hormone tests and pelvic ultrasound	Pelvic ultrasound	-	Hysteroscopy	-	-	-

Y= years; M= months

**Figure 1:**
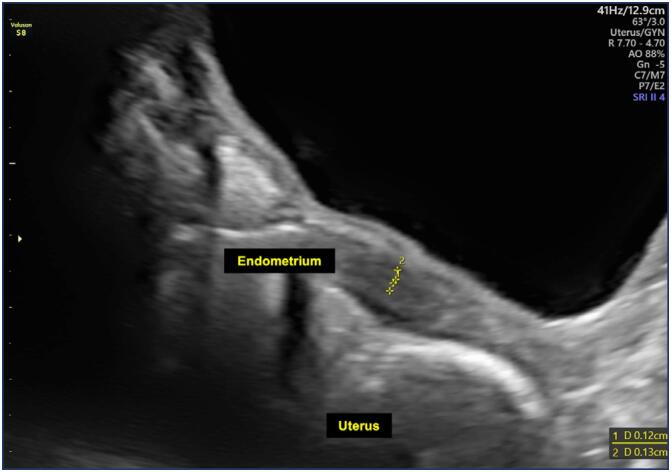
Pelvic ultrasound image showing a uterus with prepubertal dimensions and an endometrial thickness of 1.2 mm, indicated by the dashed line (Ultrasound device - Voluson S8 – GE Health Care)

At the age of 10 years and 2 months, complementary examinations revealed estradiol levels of 20.5 pg/mL (prepubertal <27 pg/mL), LH <0.07 mIU/mL (prepubertal <0.3 mIU/mL), and FSH <0.8 mIU/mL (prepubertal <2.1 mIU/mL). Prolactin was slightly elevated at 11 ng/mL (reference range for children: 4–8 ng/mL), while thyroid-stimulating hormone (TSH) was normal (2.1 μIU/mL). The abdominal pelvic ultrasound revealed the uterus with prepubertal characteristics, including a uterine body length of 13 mm, volume of 6.1 cm³, cervical length of 9 mm, and an endometrial thickness of 1.2 mm, with a prepubertal body/cervix ratio = 1 (Figure 1). The right and left ovaries measured 3.7 cm³ and 3.9 cm³, respectively, with the presence of antral follicles. Radiographic bone age was estimated at 12 years (SD = 11.7m), indicating an advancement compared to chronological age, suggesting an acceleration in the development process.

**Figure 2:**
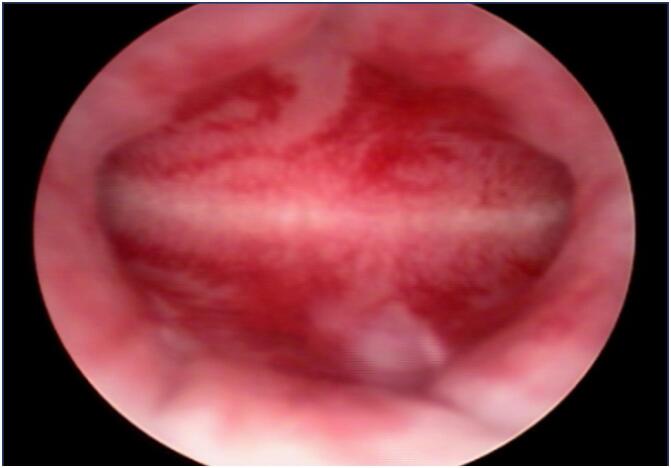
Hysteroscopy image of the uterus with initial proliferative endometrium finding

**Figure 3:**
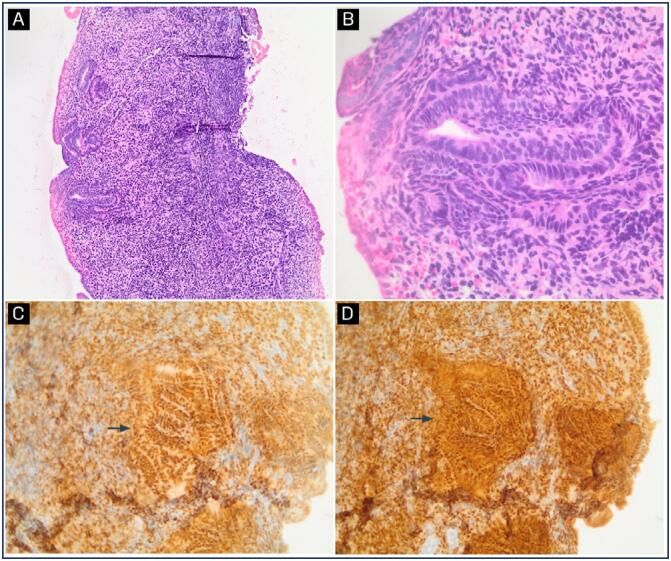
Histological sections of endometrium

Axillary odor was noted at 10 years and 8 months (Table 1). Given the persistence of vaginal bleeding without complete pubertal maturation, hysteroscopy and vaginoscopy were performed at 10 years and 10 months under sedation to investigate potential structural causes. Hysteroscopy was performed by a vaginoscopy approach, utilizing saline solution as the distension media. Under sedation, the patient was placed in gynecologic position; a 4mm Bettocchi hysteroscope with a direction of view of 30 degrees and 3,6 mm diameter (Karl Storz, Tuttlingen, Germany) was introduced through the intact hymenal orifice. Biopsy was performed under direct visualization with a 5 French biopsy and grasping forceps through the Bettocchi inner sheath. The examination revealed a vagina with smooth mucosa, a flattened cervix, and a small uterine cavity, all compatible with prepubertal characteristics and an early proliferative endometrium (Figure 2). Histopathological analysis of the endometrial biopsy showed proliferative endometrium with positive estrogen and progesterone receptors (Figure 3), suggesting hypersensitivity of the endometrium to estrogenic stimuli.

(A) Endometrial section showing tubular glands with regular spacing (hematoxylin and eosin stain, 100x) (B) Glands lined with pseudostratified columnar cells, no atypia (hematoxylin and eosin stain, 400x). (C) The arrow points to a positive nuclear marker for estrogen receptor (200x). (D) The arrow points to a positive nuclear marker for progesterone receptor (200x)

Thirty days after the hysteroscopy, at 10 years and 11 months, the patient exhibited breast development, reaching Tanner M2P2 (Table 1). At 11 years and 2 months, a new evaluation showed no difference in the bone age in comparison to the previous exam (SD = 11. 9m), and, at her last appointment, at 11 years and 9 months, the patient had M3P3 and menstrual irregularity, with episodes occurring every two to three months.

## Discussion

This case report describes a young girl who presented with vaginal bleeding at the age of 8 years in the absence of secondary sexual characteristics. At the age of 10 years, a proliferative phase endometrium was confirmed by histopathologic analysis, despite clearly prepubertal gonadotropin levels. The bleeding was likely of uterine origin, supported by hysteroscopic assessment and endometrial biopsy. Isolated menarche is an uncommon condition,^(14)^ whose etiology and natural history remain poorly understood.^(15,16)^ However, our case does not comply with the criteria for central precocious puberty, which is defined by the appearance of secondary sexual characteristics before the age of 8 years in girls.^(3)^

In our case, the histopathological analysis revealed positive estrogen and progesterone receptors, indicating that the endometrium had been responsive to estrogenic stimulation. Other case reports infer that the cause for isolated menarche is the hypersensitivity of the endometrium to very low levels of estrogens, similar to increased breast sensitivity to estrogens in premature thelarche.^(11,13)^ Endometrial proliferation is triggered by a basal concentration of estrogen in the plasma at the early follicular phase with a plato of 50 pg/mL.^(17)^ Therefore, it is possible that the patient may have reached this threshold locally, even though the abdominal pelvic ultrasound did not show endometrial thickening, which would have corroborated with this hypothesis.^(13)^ Even so, the histopathological analysis confirmed the estrogenic action, as demonstrated by the presence of both estrogen and progesterone receptors.

It is known that endocrine disrupting chemicals (EDCs) can interfere with female fertility, just as the experimental study by Lavogina et al.^(18)^ showed in vitro, with primary endometrial stromal cells from healthy women in the proliferative phase. The group treated the cells for nine days with or without EDCs in different concentrations. They proved that at least half of the EDCs studied promoted the decidualization of endometrial cells.^(18)^ Although no direct evidence of EDC exposure was found in this case, their potential role in prepubertal endometrial stimulation remains a plausible environmental factor.^(11)^

Endocrine causes of vaginal bleeding include hormonal withdrawal, isolated premature menarche, central or peripheral precocious puberty, and primary hypothyroidism. In such cases, bone age advancement is typically accompanied by other pubertal signs, such as breast development, pubic hair, accelerated growth, or the presence of ovarian cysts.^(19)^ In previous reports, bone age was found to be normal or delayed for chronological age in most patients with isolated menarche, reflecting the absence of a sustained estrogenic effect.^(10)^ However, our patient presented advanced bone age in the absence of secondary sexual characteristics or ovarian enlargement at the time of initial bleeding. Our findings suggest that exposure to low levels of estrogen can stimulate bone maturation, as proposed by Cutler^(20)^

In this case report, the patient presents dysautonomic syndrome, which refers to dysfunction of the sympathetic and/or parasympathetic branches of the autonomic nervous system.^(21)^ A literature review did not reveal any relationship between this syndrome and menstrual disorders. However, the term "dysautonomia" is broadly used to describe a range of autonomic impairments,^(22)^ including familial dysautonomia, a rare autosomal inherited disease that affects the autonomic, sensory, and motor nervous systems.^(23)^ In women with familial dysautonomia, menarche is often delayed, and monthly hormonal fluctuations may be sufficient to disturb autonomic homeostasis and trigger dysautonomic crisis. Nonetheless, plasma sex hormone levels are typically within normal ranges in these women, possibly indicating that the defect in the autonomic nervous system does not affect sex hormone production or secretion.^(23)^

The patient had been using CoQ10 for the treatment of dysautonomic syndrome since she was 6 years old. CoQ10 is a lipophilic coenzyme essential for all cell types, as it is intrinsically involved in electron transport in the mitochondrial respiratory chain. In the clinical context, CoQ10 supplementation is beneficial for cardiovascular and neurodegenerative diseases.^(24,25)^ Although a relationship between CoQ10 and estrogen receptor activation has not been clearly established in the literature, our case raises the question of an indirect mechanism of local hormonal modulation.

In this context, some studies have shown that CoQ10 supplementation can significantly increase serum testosterone levels in men and birds.^(26,27)^ In theca cells, LH stimulation promotes testosterone production, while in granulosa cells, testosterone is converted into estradiol under FSH stimulation,^(28)^ suggesting that CoQ10 might indirectly enhance local estrogen production by increasing androgen availability. This mechanism could potentially contribute to local estrogenic stimulation and the activation of endometrial estrogen and progesterone receptors, since estrogen promotes progesterone receptor expression.^(29)^ Although serum estradiol levels remained within prepubertal ranges in our patient, the histopathological analysis supports a localized hormonal effect sufficient to induce receptor expression due to endometrial hypersensitivity. Conversely, CoQ10 supplementation has also been associated with lower plasma levels of testosterone, estrogen, and progesterone in rats,^(30,31)^ although it has been shown to restore estradiol levels following treatment in obese female rats.^(32)^

Although this case offers valuable insights, there is one limitation to consider in our study. The absence of long-term follow-up data prevents a full understanding of its effects on the patient's reproductive health and final pubertal outcome. When test results are normal, annual follow-up is recommended to monitor pubertal development and to offer support to the girl and her family.^(13)^ However previous studies by Blanco-Garcia et al.,^(16)^ and Murram et al.^(33)^ based on small case series, suggest that isolated menarche alone does not negatively impact final height or fertility. These studies indicate that secondary sexual characteristics and full maturation of the HPG axis tend to develop gradually over time.

To our knowledge, this is the first case report describing the use of endometrial biopsy by hysteroscopy in a prepubertal girl with recurrent vaginal bleeding. This approach was important to visualize the endometrium and confirm its activity through the presence of estrogen and progesterone receptors. Similar reports rely only on hormonal tests, bone age evaluation, and pelvic ultrasound.^(8,10,16,34)^ Pelvic ultrasound is essential to establish normal anatomy and exclude tumors, and hormonal profile is necessary to exclude precocious puberty.^(13)^

Our observations suggest that hormone levels and ultrasound alone may not be sufficient to establish a diagnosis in cases of prepubertal vaginal bleeding, since the literature reports that these patients often show no gonadotropin-releasing hormone response, no elevated estradiol levels, and normal pelvic ultrasound results.^(35)^ In these situations, examination under anesthesia may help rule out vaginal or cervical lesions, foreign bodies, or infections.^(13)^ In cases of recurrent bleeding with normal hormone and imaging results, additional procedures such as vaginoscopy and endometrial biopsy by hysteroscopy may be necessary.^(14,35)^ However, these exams are recommended only when the cause of prepubertal bleeding remains unclear, highlighting the need to evaluate each case individually before moving forward with invasive procedures.^(19)^

## Conclusion

This is the first case report demonstrating the action of estrogen on the endometrium of a girl without the development of secondary sexual characteristics, confirmed by histopathologic analysis showing estrogen and progesterone receptor expression. This case emphasizes the importance of considering isolated vaginal bleeding in prepubertal girls as a possible manifestation of endometrial hypersensitivity to low estrogen levels, which may alter the usual chronology of pubertal development. In addition, this is the first report to include endometrial biopsy via hysteroscopy in this setting, providing additional information beyond what hormone testing and pelvic ultrasound alone can provide. The results suggest a possible local hormonal modulation, raising the question of whether prolonged use of CoQ10 supplementation may have contributed to this phenomenon, and highlight the importance of personalized evaluation in cases of unexplained prepubertal vaginal bleeding.

## Data availability

: The authors did not make the data from this article available in repositories prior to submission.
